# Iron Metabolism, Ferroptosis, and the Links With Alzheimer’s Disease

**DOI:** 10.3389/fnins.2019.01443

**Published:** 2020-01-29

**Authors:** Nao Yan, JunJian Zhang

**Affiliations:** Department of Neurology, Zhongnan Hospital of Wuhan University, Wuhan, China

**Keywords:** iron metabolism, iron overload, ferroptosis, Alzheimer’s disease, reactive oxygen species

## Abstract

Iron is an essential transition metal for numerous biologic processes in mammals. Iron metabolism is regulated via several coordination mechanisms including absorption, utilization, recycling, and storage. Iron dyshomeostasis can result in intracellular iron retention, thereby damaging cells, tissues, and organs through free oxygen radical generation. Numerous studies have shown that brain iron overload is involved in the pathological mechanism of neurodegenerative disease including Alzheimer’s disease (AD). However, the underlying mechanisms have not been fully elucidated. Ferroptosis, a newly defined iron-dependent form of cell death, which is distinct from apoptosis, necrosis, autophagy, and other forms of cell death, may provide us a new viewpoint. Here, we set out to summarize the current knowledge of iron metabolism and ferroptosis, and review the contributions of iron and ferroptosis to AD.

## Introduction

Iron is the second most abundant metal of the earth’s crust following aluminum and the most abundant transition metal in biology. Iron plays a crucial role in various vital biological processes by different oxidation states, including oxygen transportation, DNA synthesis and repair, respiratory activity, myelin synthesis, and cellular metabolism (Patel and Ramavataram, 2012; Levi and Taveggia, 2014; Ndayisaba et al., 2019; Xu, 2019). Iron homeostasis is maintained via multiple mechanisms such as hepcidin and iron regulatory proteins (IRPs) at the systemic and cellular levels (Pantopoulos et al., 2012). Disruption of iron homeostasis can result in excessive intracellular iron accumulation, thereby damaging proteins, lipids, and DNA via generation of free radicals and oxidative stress (Ward et al., 2014).

Accumulating studies have shown that iron dyshomeostasis is involved in the pathogenesis of Alzheimer’s disease (AD). Iron depositions in the specific brain regions have been proved in AD through imaging and histologic examinations (Altamura and Muckenthaler, 2009; Mills et al., 2010; Apostolakis and Kypraiou, 2017; Lee and Lee, 2019). Ferroptosis is a newly non-apoptotic form of cell death, which is characterized by the iron-dependent accumulation of lipid reactive oxygen species (ROS) (Dixon et al., 2012; Yan and Zhang, 2019). Recent studies show that ferroptosis plays a key role in neuronal death and neurological diseases including traumatic brain injury (TBI), stroke, Friedreich’s ataxia, AD, Parkinson’s disease (PD), and Huntington’s disease (HD) (Alim et al., 2019; Kenny et al., 2019). These studies provide us novel perspectives. Hence, we summarize the current knowledge of iron metabolism and ferroptosis, and review the contributions of iron and ferroptosis to AD.

## Brain Iron Metabolism

Iron is a vital element for a myriad of fundamental biological functions, because it can readily donate and accept electrons to participate in oxidation–reduction reactions. But it is also toxic when excess “free” iron is present. Indeed, this redox-active iron can catalyze the production of ROS by Fenton reaction, thereby attacking cellular lipids, proteins, and nucleic acids and causing cell damage (Dev and Babitt, 2017; Li et al., 2019; Wang and Babitt, 2019). Iron plays a crucial role in the synthesis of myelin and neurotransmitters in the central nervous system. However, excessive brain iron concentrations are thought to be a potential cause for various neurodegenerative diseases including AD, PD, HD, and multiple sclerosis (Ward et al., 2014). And iron deficiency in infants and the developing brain can easily cause neurological deficits and mental retardation (Chiou et al., 2019b; Thirupathi and Chang, 2019). Accordingly, iron metabolism must be elegantly regulated by a complex network of processes including absorption, utilization, recycling, and storage. This complex, highly regulated process involves a series of proteins such as ferritin (FTH1), transferrin (Tf), transferrin receptor 1 (TfR1), divalent metal transporter 1 (DMT1, SLC11A2), ferroportin (FPN1), and hepcidin (Bogdan et al., 2016).

The blood–brain barrier (BBB) is the unique structure in the brain that is different from other tissues and organs, which tightly regulates the movement of ions, molecules, and cells between the blood and the brain (Daneman and Prat, 2015). Thus, the endothelial cells of the BBB are the key site for regulating brain iron uptake, and the Tf/TfR1 pathway is the main brain iron absorption route depending on BBB (Duck et al., 2017; Chiou et al., 2019a; Qian and Ke, 2019). Research shows that Tf is the iron carrier that is responsible for delivering ferric iron to erythrocyte precursors and other tissues, but the ferrous iron must be oxidized to ferric iron by hephestin, a multi-copper ferroxidase enzyme, before it binds to Tf (Yiannikourides and Latunde-Dada, 2019). TfR1 is highly expressed on the luminal side of endothelial cells. Accordingly, Tf-bound serum iron in circulation binds to its receptor (TfR1) firstly, and Tf/TfR1 complex is taken up through endocytosis; then ferric iron is released from Tf, and ferric iron is reduced to ferrous iron by ferric reductase six-transmembrane epithelial antigen of prostate 3 (STEAP3) in the acidic endosome, thereby transferring by DMT1 for metabolic synthesis or for storage with ferritin in the cytoplasm. DMT1 is a metal transporter that principally transports iron from the endosome to cytoplasm (Skjorringe et al., 2012; Yu and Chang, 2019). Furthermore, iron could also be exported by FPN1 to the extracellular environment. FPN1 is the sole known intracellular iron exporter in mammals, which plays a vital role in the export of cellular iron. And FPN1is controlled by the iron-regulatory hormone hepcidin, which is mainly synthesized and secreted by hepatocytes (Ganz, 2013). Hepcidin binds directly to FPN1 and triggers its internalization, ubiquitination, and degradation (Sangkhae and Nemeth, 2017; Cornelissen et al., 2019). Ultimately, iron across the BBB can be absorbed by neurons, microglia, and astrocytes via the same iron metabolism-related proteins (McCarthy and Kosman, 2015; Simpson et al., 2015; Bu et al., 2019; Yan and Zhang, 2019).

But different cell types have different ways of iron absorption. Neurons and microglia can acquire iron by means of the Tf/TfR1 pathway or absorb non-Tf-bound iron (NTBI) via the luminal DMT1-dependent pathway (Ke and Ming Qian, 2003; Urrutia et al., 2013; Zarruk et al., 2015). Recent studies show that the binding of H-ferritin to the H-ferritin receptor (Tim-1/2) may be the major source of iron uptaking for oligodendrocytes (Todorich et al., 2011; Chiou et al., 2018; Qian and Ke, 2019). In addition to the Tf/TfR1 or DMT1 pathway, astrocytes may acquire iron by their end-feet processes (Biasiotto et al., 2016; Qian and Ke, 2019; Xu et al., 2019). In this way, brain iron levels are precisely regulated to participate in normal neuronal function.

## Ferroptosis and Iron

Ferroptosis is a novel defined form of regulated cell death, which is characterized by iron-dependent lipid peroxidation that ultimately leads to oxidative stress and cell death (Doll and Conrad, 2017). Ferroptotic cell death is morphologically, biochemically, and genetically distinguished from other forms of cell death including apoptosis, necrosis, autophagy, and pyroptosis (Dixon et al., 2012; Stockwell et al., 2017). In cytological changes, the chief distinguishing features of ferroptosis are decreased or vanished mitochondria cristae, condensed mitochondrial membrane, and mitochondria volume shrinkage (Angeli et al., 2017; Stockwell et al., 2017). The hallmark of ferroptosis is accumulation of iron-dependent ROS, reduction of glutathione (GSH) level, and inactivation of glutathione peroxidase 4 (GPX4); thereby this redox dyshomeostasis triggers cell death (Friedmann Angeli et al., 2014; Hirschhorn and Stockwell, 2019). Intracellular iron accumulation can generate ROS and cause oxidative stress via Fenton reaction, thereby promoting the peroxidation of proteins, nucleic acids, and lipids, which is the key process to propagate ferroptosis (Cao and Dixon, 2016; Fanzani and Poli, 2017). Interestingly, ferroptosis inducers were firstly found before the term *ferroptosis* was coined. To start with, researchers unexpectedly found that the small molecule erastin induced non-apoptotic cell death in tumorigenic cells (Dolma et al., 2003). Afterward, two ras-selective lethal small molecular compounds (RSL3 and RSL5) were screened out that induced non-apoptotic and iron-dependent oxidative cell death. And an iron chelator (desferrioxamine) and an antioxidant (vitamin E) could prevent this form of cell death, which showed similar properties with previous cell death form induced by erastin (Yang and Stockwell, 2008, 2016). Thus, ferroptosis was named for this novel non-apoptotic cell death in 2012, which suggests that redox-active iron plays a critical role in this novel cell death mechanism (Dixon et al., 2012; Mou et al., 2019).

Although the exact mechanisms of iron in the signaling pathway of ferroptosis are still poorly understand, the involvement of iron dyshomeostasis in the ferroptotic process is beyond any doubt. Indeed, lipid peroxidation and lethal ROS resulting from iron-mediated Fenton reaction or enzymatic oxygenation is the essential step of ferroptosis (Dixon and Stockwell, 2013; Xie et al., 2016; Doll and Conrad, 2017). Iron homeostasis is a complex process and relies on coordination of multiple iron metabolism proteins. Once the expression of these molecules is altered, ferroptosis may be triggered. Recent research found that the iron-carrier serum protein Tf and its cell surface receptor, TfR1, played critical roles in ferroptotic cell demise. The Tf/TfR1 pathway is mainly responsible for the absorption of cell iron. And abnormal TfR1 recycling and palmitoylation can result in neurodegeneration with brain iron accumulation (NBIA) (Gao et al., 2015; Drecourt et al., 2018; Park and Chung, 2019). The DMT1, another important iron-absorbing protein, is also closely associated with brain iron accumulation in neurodegenerative diseases. Multiple studies have shown that DMT1 overexpression contributed to iron accumulation in the substantia nigra and dopaminergic neuron loss (Salazar et al., 2008; Zhang et al., 2017; Ingrassia et al., 2019). Ferritin is the main intracellular iron storage protein composed of FTL1 (light chains) and FTH1 (ferritin heavy), which preserves excess iron in a redox inactive form and prevents the cell and tissue from oxidative damage (Theil, 2013; Dowdle et al., 2014; Lal, 2019). Nuclear receptor coactivator 4 (NCOA4) is a selective cargo receptor, which is responsible for binding to ferritin and transporting it to the lysosome for degradation (Mancias et al., 2014). Thus, this process is termed ferritinophagy, and NCOA4-mediated ferritinophagy induces ferroptosis by degradation of ferritin and increasing cellular labile iron levels (Gao et al., 2016; Hou et al., 2016; Quiles Del Rey and Mancias, 2019). Indeed, abnormal iron balance caused by dysfunctional ferritinophagy is critical to induce ferroptosis and also plays a central role in neurodegenerative diseases mediated by ferroptosis (Tang et al., 2018). The mechanisms of iron overload in ferroptosis are not well understood partly because of the difficulty of measurement. Investigators currently have designed a unique fluorescence resonance energy transfer (FRET) probe, FRET Iron Probe 1 (FIP-1), which provides direct evidence for changes in labile iron status during ferroptosis (Aron et al., 2016).

Furthermore, iron is also an important component that composes a subunit of oxidase for lipid peroxidation. Lipoxygenases (LOXs) are a family of non-heme iron enzymes, which can drive ferroptosis by peroxidation of cellular membrane polyunsaturated fatty acids (PUFAs). The iron in LOX active sites plays an important role in generating toxic lipid hydroperoxides. And the iron chelators (deferoxamine and deferiprone) also could rescue ferroptosis through removing the essential catalytic iron from LOXs (Abdalkader et al., 2018; Zhou et al., 2019). Recent studies show that arachidonate-15-lipoxygenase (ALOX15) plays a significant role in erastin-induced ferroptosis by facilitating the formation of lipid peroxides (Kagan et al., 2017; Shintoku et al., 2017). And intracellular iron accumulation and lipid peroxidation are two key events initiating ferroptosis (Vanden Berghe et al., 2014). Iron not only directly catalyzes the formation of ROS, but also synthesizes the LOXs that oxidize PUFAs to result in lipid peroxides in ferroptosis. Altogether, iron is the essential component of ferroptosis (Stoyanovsky et al., 2019).

## Iron Metabolism Linking With AD Pathology

Alzheimer’s disease is considered currently as a complicated neurodegenerative disease with multiple cerebral pathologies. The most typical histopathological features of AD are the deposition of extracellular amyloid-β (Aβ) in senile plaques (SPs) and intracellular neurofibrillary tangles (NFTs) formed by hyperphosphorylation of tau protein. These changes of nerve cells are ultimately accompanied by dead and dying neurons (Belaidi and Bush, 2016; Lane et al., 2018). Many studies have shown that brain iron dyshomeostasis is tightly associated with Aβ plaques and NFTs. Indeed, iron deposition has been involved in the two misfolding process. SP and NFT complexes cover redox-active transition metals (Sayre et al., 2000; Weinreb et al., 2016).

Aβ precursor protein (APP) is a type 1 transmembrane glycoprotein, which is the crucial precursor to the production of Aβ. APP can undergo proteolytic cleavage first by α-secretase or β-secretase and then γ-secretase. In health, α-secretase firstly cleaving APP means being on the non-amyloidogenic pathway. However, once APP is cleaved first by the β-secretase enzyme, it finally can produce the neurotoxic 40- to 42-amino-acid amyloid (the amyloidogenic pathway). And furin plays a key role in mediating the proteolytic activation ratio of α-secretase or β-secretase. Indeed, the concentration of furin protein was positively correlated with α-secretase activity and negatively correlated with β-secretase activity (Guillemot et al., 2013). Excessive intracellular iron concentration is responsible for reducing transcriptional regulation of furin. And iron can reinforce the β-secretase activity by the decrease of furin protein, thereby directly increasing Aβ production by the amyloidogenic pathway (Ward et al., 2014). Furthermore, APP mRNA encodes iron-responsive elements (IREs) in the 5’-untranslated region (5’-UTR mRNA), which is closely related to intracellular iron content (Rogers et al., 2008). When the intracellular iron concentrations increase, it upregulates the translation of APP by virtue of IREs in the 5’-UTR mRNA, thereby increasing the amount of APP protein and potentially producing more Aβ (Becerril-Ortega et al., 2014; Peters et al., 2015; Telling et al., 2017). Additionally, iron also directly binds to Aβ in His6, His13, and His14 amino acid residues, thereby strengthening the neurotoxicity of Aβ (Uranga and Salvador, 2018; Wojtunik-Kulesza et al., 2019).

Indeed, it is widely accepted that APP also physically interacts with the cell surface FPN1 through stabilizing and locating FPN1, and enabling intracellular iron efflux. Recent research indicates that brain FPN1 levels reduce brain iron accumulation in APP-KO mice, and the ability of cerebral iron export was significantly decreased, which reveals that APP/FPN1 plays a crucial role in modulating cerebral iron homeostasis (Belaidi et al., 2018). New research shows that post-translational modulation plays a key role in locating and trafficking of APP to the cell surface. The study confirmed that damaged *N*-glycosylation or phosphorylation of APP impeded the trafficking of APP to the cell surface, which disturbed FPN1 stability and reduced cell surface FPN1 levels, thereby altering neuronal iron homeostasis and resulting in intracellular iron retention. Meanwhile, Post-translational modifications of APP induced conformation changes and altered the cleavage preference of each secretase, thereby dysregulating APP processing and Aβ generation (Wang et al., 2017; Tsatsanis et al., 2019). Indeed, there is also a study that shows that iron accumulation induces binding of APP to β-secretase, thereby resulting in Aβ accumulation (the amyloidogenic pathway), and reduces the affinity of APP/FPN1 mediating iron export in microglia (Gong et al., 2019). Furthermore, synchrotron X-ray spectromicroscopy technology also shed light on the presence of ferrous iron (redox-active iron phases) in amyloid plaque cores. The research indicates that Aβ plays a important role in reducing ferric iron into ferrous forms; this transformation process leads to excess free radical generation and inducing neuronal damage, which could promote the understanding of the role of iron in the pathology of AD (Everett et al., 2018).

Neuroinflammatory response and microglial activation also are the typical changes of AD pathogenesis. Recent studies have shown that iron accumulation in microglia also contributes to microglial dysfunction and Aβ accumulation. In APP/PS1 mice, a commonly used animal model of AD, which overexpresses the APP and presenilin 1 (PS1), the researcher attests that iron accumulation can drive microglia to switch to a glycolytic metabolic, thereby reducing the capacity to phagocytose Aβ, ultimately leading to Aβ accumulation (McIntosh et al., 2019). And microglia has two polarization states with opposite functions including M1 proinflammatory, cytotoxic cell type and anti-inflammatory, prorepair M2 cell type. M1 phenotype microglia plays an important role in the pro-inflammatory response. Recent research shows that iron accumulation can drive microglia polarization to M1 phenotype. And it was found out that there is a large amount of activated iron-containing microglia around Aβ plaques (Kroner et al., 2014; van Duijn et al., 2017). Additionally, cortical iron deposition is increasingly recognized as a novel imaging marker for AD diagnosis through using high field magnetic resonance imaging (MRI) to scan hemispheres of AD patients (Peters et al., 2015; van Duijn et al., 2017; Bulk et al., 2018; Kenkhuis et al., 2019). And cerebral quantitative susceptibility mapping (QSM), an MRI method sensitive to brain iron, reveals that the brain iron burden is elevated in AD patients, combined with Aβ positron emission tomography (PET), which indicates that brain iron load is positively associated with Aβ deposition-related cognitive decline, suggesting that iron may combine with Aβ to exacerbate the cognitive function damage. The pathologic mechanism could be that iron promotes the production of free radicals and oxidative stress and possibly also involves ferroptosis (Ayton et al., 2017b; van Bergen et al., 2018). And other research validates that spatial colocalization of cerebral iron with Aβ plaques increased the risk for AD dementia (van Bergen et al., 2016). Moreover, in mild and moderate AD patients, the researchers determined the magnetic susceptibility values of deep gray matter nuclei with QSM and evaluated the cognitive functions through Montreal cognitive assessment (MoCA) and mini-mental state examination (MMSE). They found that the magnetic susceptibility of the left caudate nucleus was significantly correlated with the severity of cognitive questionnaire scores (MMSE and MoCA) in AD (Du et al., 2018). Altogether, these studies indicate that it is possible for brain iron levels quantified by QSM to be a biomarker of the severity of AD.

Ferritin is the major iron storage protein, and cerebrospinal fluid (CSF) ferritin could reflect cerebral iron levels. Studies have shown that CSF ferritin can be recognized as a potential biomarker, which is better to predict disease progression and future cognitive impairment, especially in patients with high Aβ deposition or APOE-ε4 carriers. And high CSF ferritin levels in the presence of Aβ accumulation indicate a high risk of brain hypometabolism and cognitive decline. It is generally accepted that APOE-ε4 is the greatest genetic risk for AD. And research shows that APOE-4 could intervene in brain iron homeostasis by increasing ferritin levels, thereby strengthening the susceptibility to AD. These findings also indicate that CSF ferritin has the potential to be a biomarker of AD and highlight the role of iron in the AD pathological mechanism (Ayton et al., 2015, 2017a; Ayton et al., 2018; Diouf et al., 2019).

Under physiological conditions, tau is a microtubule-associated protein that supports neuronal microtubule structure, which plays a key role in axonal transport, protein trafficking, and cognitive function (Wang and Mandelkow, 2016; Joppe et al., 2019). However, tau hyperphosphorylation aggregates into NFTs, which is a major pathological hallmark of AD. And it is widely believed that brain iron dyshomeostasis is closely associated with the formation of NFTs and the progression of tau-mediated neurodegeneration. Iron not only can regulate tau phosphorylation but also can induce the aggregation of hyperphosphorylated tau (Kim et al., 2018; Rao and Adlard, 2018). Indeed, iron deposition is colocalized with NFTs in a special brain region associated with the progression of neurodegeneration (Rao and Adlard, 2018). Furthermore, many proline-directed protein kinases are also closely interrelated with tau phosphorylation in AD such as glycogen synthase kinase-3 (GSK3), cyclin-dependent protein kinase-5 (CDK5), and mitogen-activated protein kinases (MAPKs) (Jouanne et al., 2017). Indeed, excess neuronal iron can promote tau hyperphosphorylation and facilitate the formation of NFTs through cyclin-dependent kinase (CDK5)/P25 complex and GSK-3β kinase pathways (Guo et al., 2013; Vossel et al., 2015; Kim et al., 2018; Wang et al., 2019). Additionally, the study also shows that dysfunctional insulin signaling is associated with iron-induced abnormal phosphorylation of tau in AD (Wan et al., 2019).

Research shows that tau plays an important role in protein trafficking via stabilizing neuronal microtubules. And tau can mediate cellular iron efflux through trafficking APP to the cell surface in normal physiology (Lei et al., 2012; Wang and Mandelkow, 2016). Tau hyperphosphorylation and aggregation impair surface trafficking of APP, thereby contributing to toxic neuronal iron accumulation and aggravating NFTs, which leads to a vicious cycle (Wong et al., 2014; Ndayisaba et al., 2019). Indeed, the tau-mediated APP pathway can prevent ferroptotic damage by reducing iron-mediated neurotoxicity in a transient middle cerebral artery occlusion (MCAO) rat model (Tuo et al., 2017). Therefore, iron- and tau-mediated generation of ROS is an important mechanism of neuron damage and death.

## Ferroptosis Linking With AD

Aging is one of the greatest risk factors for the disease. And iron accumulates in the brain with aging processes; brain iron deposition is more serious in special regions in neurodegenerative diseases associated with oxidative stress and neuronal death (Hare et al., 2013; Ward et al., 2014). Indeed, brain iron dysregulation along with reduced endogenous antioxidant systems including GPX is closely linked to AD pathology. The level of brain iron is positively correlated with AD progression and cognitive decline (Ayton et al., 2017b; Derry and Kent, 2017). Patients with mild cognitive impairment accompanying high Aβ plaque load showed higher cortical iron, which increased the risk of AD (Hare et al., 2013; van Bergen et al., 2016; Lupton et al., 2017).

The brain is more susceptible to oxidative damage than other tissues owing to high demand for dynamic energy metabolism. And neurons are less tolerant of oxidative stress due to a low antioxidant defense system (Dringen, 2000). The progressive loss of neurons is a direct cause of clinical symptoms correlating with AD. And oxidative stress is an essential pathological mechanism of AD (Thapa and Carroll, 2017; Cobley et al., 2018). Iron is an essential cofactor for metabolic reactions, but iron also can generate ROS and causes oxidative stress in the same microenvironment. Indeed, iron-induced oxidative stress directly causes destructive DNA, lipid, and protein damage, thereby leading to cell death (Liu et al., 2018). Recent studies also found that iron plays a key role in ferroptosis, a newly defined non-apoptotic cell death. Lipid peroxidation and iron dyshomeostasis and accumulation, two essential conditions of ferroptosis, have long been noted in AD brains. That ferroptosis may contribute to the neuronal loss of AD attracts more and more attention (Masaldan et al., 2019). Ferroptosis, a novel form of cell death characterized by intracellular iron overload, may provide us a new perspective to understand the pathological mechanism of AD (Weiland et al., 2018; Nikseresht et al., 2019).

Glutathione peroxidase 4 is a unique anti-peroxidant enzyme, which inhibits lipid peroxidation through directly reducing membrane lipid hydroperoxides to lipid alcohols (Cozza et al., 2017). And GPX4 is considered as the central regulator of ferroptosis (Imai et al., 2017). Indeed, multiple studies showed that lipid peroxidation and depletion or reduction of GSH levels, the other signature of ferroptosis, also occur in AD. Recent studies revealed that hippocampi’s and frontal cortices’ GSH levels have the potential to be a predictive biomarker for AD and MCI (Mandal et al., 2015; Ayton et al., 2019). The research shows that ablation of GPX4 induces ferroptosis of spinal motor neurons, thereby resulting in rapid onset and progression of paralysis and death in adult mice. Further research shows that ferroptosis triggers the major cell death in hippocampal neurodegeneration by ablation of forebrain neuron GPX4, which directly relates to cognitive impairment. And the hallmarks of ferroptosis (iron dysregulation, lipid peroxidation, inflammation) are recognized as important preclinical signs of AD and cognitive impairment (Chen et al., 2015; Hambright et al., 2017). In addition, research shows that α-lipoic acid (LA) administration could block tau-induced iron overload, lipid peroxidation, and inflammation, which are involved in ferroptosis (Zhang et al., 2018). Iron interacts with Aβ and tau through the deposition of iron and the formation of a peptide–hemin complex, thereby participating in generating ROS that possibly involves the ferroptotic death pathway (Derry et al., 2019). Taken together, although ferroptosis in AD is unproven, it is gradually attracting more and more attention.

## Conclusion

Alzheimer’s disease is characterized by progressive cortical and hippocampal neuronal dysfunction and death; the major hypothesis mechanisms are Aβ depositions in SPs and NFTs of hyperphosphorylated tau protein (Dugger and Dickson, 2017; Roubroeks et al., 2017). However, a large number of drug clinical trials based on these two hypotheses worldwide have failed; there is still no effective way to treat it. Furthermore, the two hypotheses are subject to a growing challenge (Liu et al., 2019; Nikseresht et al., 2019). Indeed, it has been widely accepted that iron participates in the pathogenesis of AD. Iron not only aggravates toxic Aβ and hyperphosphorylated tau aggregation but also directly induces neuronal oxidative damage (Thirupathi and Chang, 2019). Considering the particularity and importance of iron in ferroptosis and the pathomechanism of AD, ferroptosis may provide a new insight into the molecular pathophysiology of the disease (Morris et al., 2018; Leong et al., 2019). Thus, future research aimed at validating the role of ferroptosis in AD is needed.

## Author Contributions

NY drafted the manuscript. JZ revised the manuscript. Both authors read and approved the final manuscript.

## Conflict of Interest

The authors declare that the research was conducted in the absence of any commercial or financial relationships that could be construed as a potential conflict of interest.
